# Mucinous cystic neoplasm of the liver with biliary communication: case report and surgical therapeutic option

**DOI:** 10.1186/s12893-020-01003-3

**Published:** 2020-12-11

**Authors:** Raphaella Ferreira, Phillipe Abreu, Vagner Birk Jeismann, Vanderlei Segatelli, Fabricio Ferreira Coelho, Andre Ibrahim David

**Affiliations:** 1 Division of Liver Surgery, Santa Casa of Sao Paulo School of Medical Sciences, São Paulo, Brazil; 2 Department of Surgery, Jackson Memorial Hospital, Miami Transplant Institute, University of Mami, 1801 NW 9th Ave, 7th Floor, Miami, FL 33137 USA; 3 Division of Digestive Surgery, Department of Gastroenterology, University of Sao Paulo School of Medicine, São Paulo, Brazil; 4 Division of Clinical Pathology, Albert Einstein Israelite Hospital, São Paulo, Brazil; 5 Division of Liver Transplant Surgery, Samaritano Higienopolis Hospital, São Paulo, Brazil

**Keywords:** Liver neoplasm, Mucinous cystadenoma, Hepatectomy, Benign liver tumors

## Abstract

**Background:**

Mucinous cyst neoplasm of the liver (MCN-L) comprise less than 5% of all cystic liver lesions and is characterized by the presence of ovarian stroma and absence of bile duct communication.

**Case presentation:**

Here, we discuss a 45-year-old woman who presented with symptomatic liver mass. Diagnostic workup detected a 4.2 × 3.6 cm septate cyst located in segments I, V, and VIII of the liver in communication with the right hepatic duct. An open right liver resection with total bile duct excision and hilar lymphadenectomy was performed. Pathology revealed a multiloculated cyst with lined mucinous epithelium and ovarian-like stroma, consistent with low-grade MCN-L.

**Conclusions:**

This case shows that unusual location and bile duct communication can be present in MCN-L.

## Background

Cystic liver disease affects 5–10% of the world population [1]. Initially, they were termed as biliary cystadenoma and cystadenocarcinoma [1, 2]. Recently, World Health Organization (WHO) had classified the cystic neoplasm of the liver into mucinous cystic neoplasm of the liver (MCN-L) and intraductal papillary neoplasm of the bile duct (IPN-B), similar to the classification used in pancreas [1]. MCN-L comprise less than 5% of all cystic liver diseases and is characterized by the presence of ovarian stroma and absence of bile duct communication.

MCN-Ls have a female predominance, presenting with relatively nonspecific abdominal symptoms and are usually an incidental finding on imaging, since laboratory values are often normal [3]. Of those that do experience symptoms, the typical presentation includes abdominal pain, distension, nausea, and vomiting. Rarely, MCN-Ls may present from symptoms secondary to obstructive jaundice, cholangitis, intra-cystic hemorrhage, or cyst rupture [4].

Radiologically, this neoplasm is a solitary multiloculated cystic lesion, typically centrally located (mainly in segment IV), with slow-growth [5]. Bile duct communication is a feature against the diagnose of MCN-L, according to current diagnostic criteria [2]. Complete surgical resection is the management of choice for MCN-Ls, given the risk of malignant transformation and recurrence [6].

## Case presentation

A 45-year-old-female with no significant past medical history other than grade I overweight (BMI 33.6 kg/m^2^), presented with upper abdominal pain, anorexia, malaise, mild jaundice and pruritus for 6 months. The liver function tests were normal, and CA 19–9 was slightly elevated (37 U/ml).

**Fig. 1:**
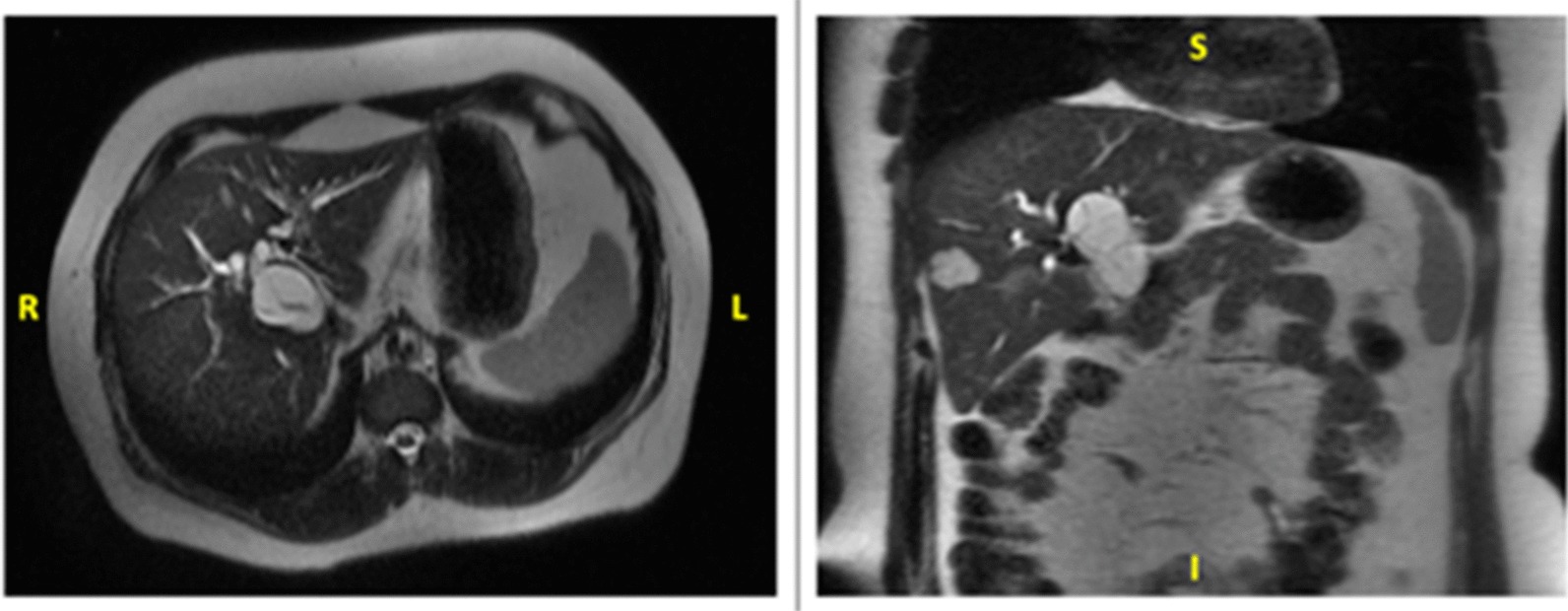
Abdominal MRI showing a hypodense lesion in segment V/VIII of the liver with few internal septations (axial and coronal planes)

Abdominal contrasted computed tomography (CT) scan demonstrated a 4.2 × 3.6 cm multiloculated cystic lesion within segment I, V and VIII of the liver, communicating with the right hepatic duct. The biliary system was dilated with thickening of the common hepatic duct, and extrinsic compression of the biliary confluence. The image also revealed a hepatic lesion in segments V/VIII, suggestive of hemangioma. Radiologic findings were confirmed by magnetic resonance imaging (MRI) (Fig. 1).

After multidisciplinary discussion, the patient was considered to present with a resectable complex cystic lesion, compatible with IPN-B associated to cholangiocarcinoma. Anatomic right hepatectomy and hilar lymphadenectomy was the proposed procedure for surgical resection. The tumor was involving the bifurcation of the main biliary hepatic ducts (right and left), then a decision was made not to submit the patient to an endoscopic retrograde cholangiography procedure due to its invasiveness characteristics and complications associated to the papillotomy, since it would not change the surgical plan at that moment in time.

**Fig. 2:**
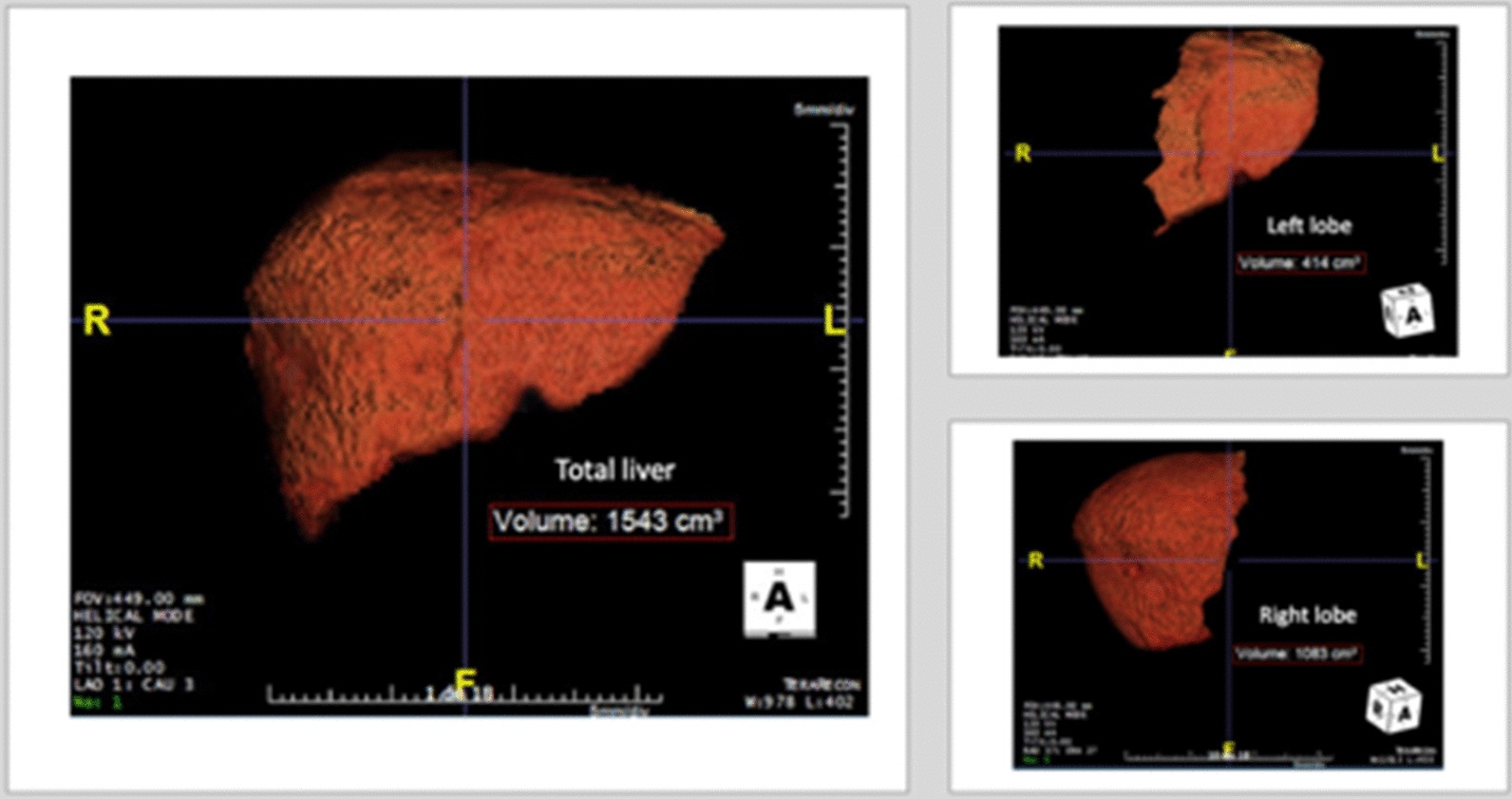
Preoperative volumetric liver assessment

**Fig. 3:**
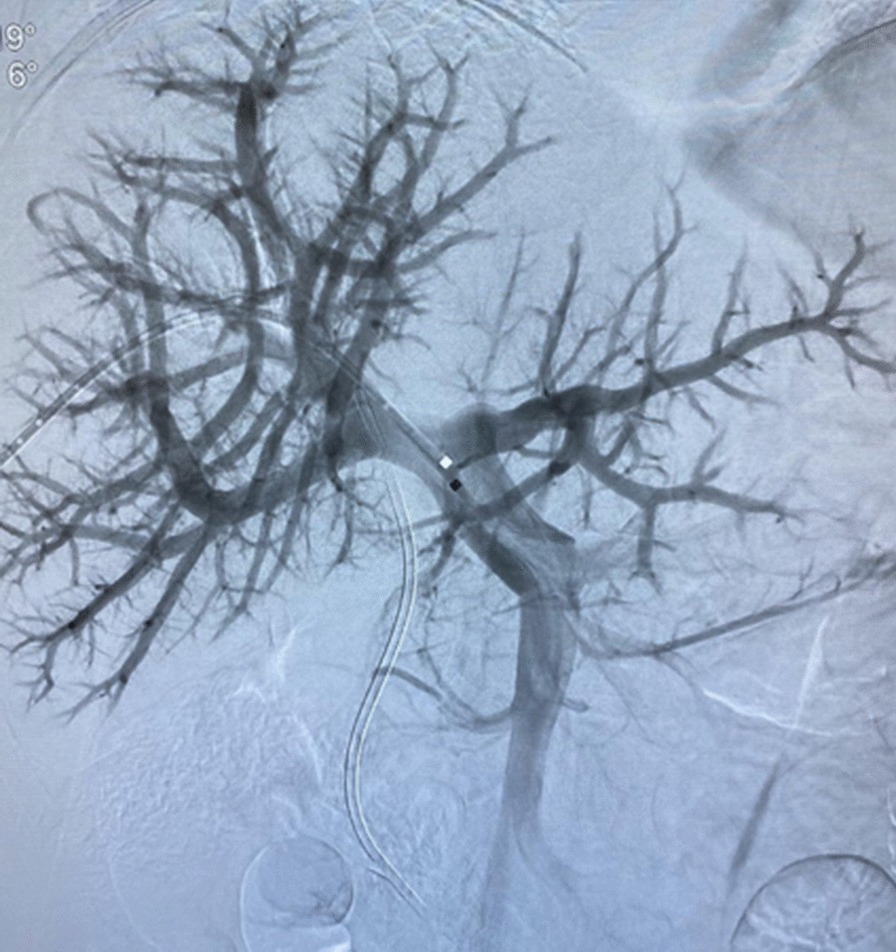
Portal vein anatomy prior to right portal vein embolization

**Fig. 4:**
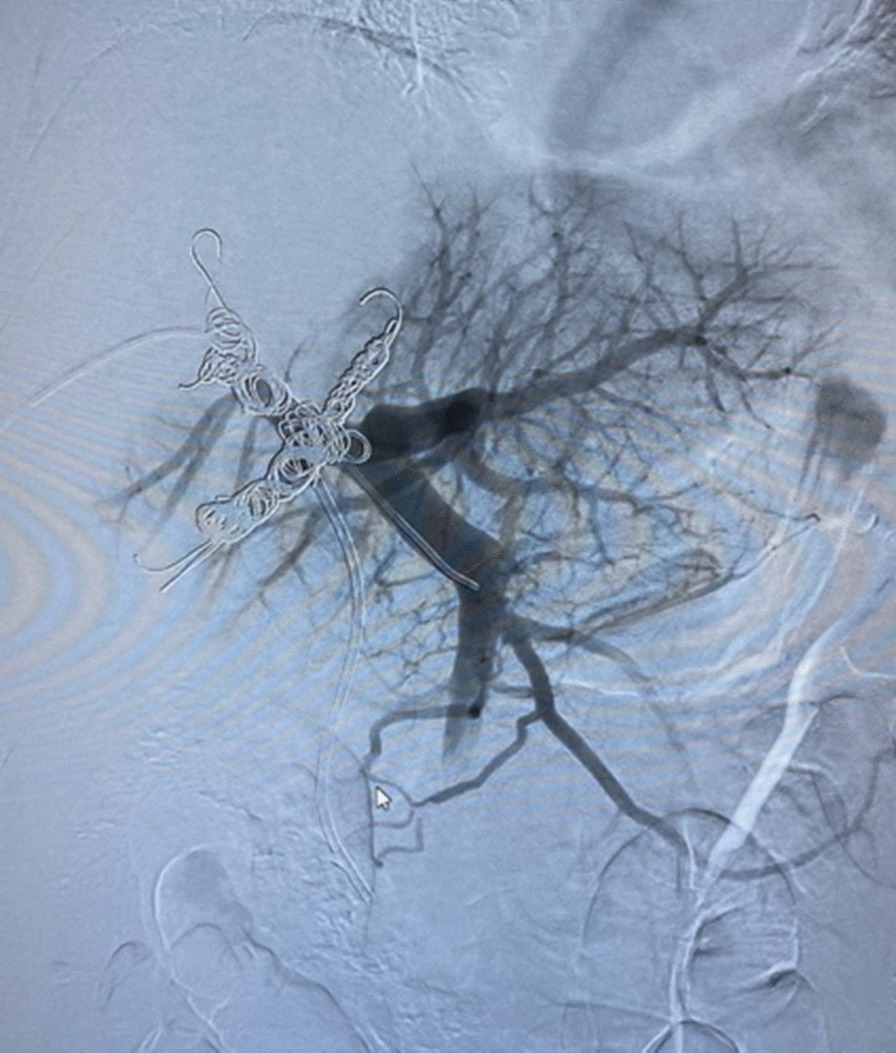
Right portal vein embolization (final aspect)

Preoperative liver volumetric assessment was performed to estimate the future liver remnant (FLR) (Fig. 2). Total liver volume (TLV) was 1543 ml, and the FLR was 414 ml (FLR/TLV = 26.8%), the ratio FLR/BW was 0.48%. Due to insufficient estimated FLR the patient was submitted to right portal vein embolization, targeting the inflow to the liver lesion (Figs. 3, 4). After 4 weeks, the estimated FLR increased to 790 ml (FLR/TLV = 45.4%; FLR/BW = 0.91%).

The abdominal cavity was assessed thought a J-shape incision. No unexpected peritoneal seeding or metastasis were found. A 4 cm cystic lesion in segments I, IV and VIII was observed. Intraoperative cholangiography through the cystic duct confirmed communication of the cyst with the bile ducts and extrinsic biliary compression. The intraoperative ultrasound assessment confirmed no macrovascular invasion, with cystic biliary communication to the right hepatic duct.

After defining the resectability, complete skeletonization of the hepatoduodenal ligament was performed. Thereafter, the distal common bile duct was isolated and divided at the level of the intrapancreatic portion, and the remainder was sent for frozen biopsy (negative). Hilar lymphadenectomy included stations 12, 8a and 13 with en-block of extra-hepatic and biliary confluence resection was performed. The right hepatic artery and right portal branch were ligated and divided, with careful dissection not to damage the vascular inflow to the FLR.

Subsequently, the liver was mobilized by dividing all ligamentous attachments. Anatomic right hepatectomy including the caudate lobe (segments I, V, VI, VII, and VIII) was performed using ultrasonic harmonic scalpel device (Ultracision™) along with the demarcation line marked by the ischemic color change of the liver surface. The left intrahepatic bile duct was resected at its origin in the umbilical fissure.

Left sided bile duct was reconstructed after removal of the specimen from the abdominal cavity. The Roux limb was placed up in a retro colic fashion, and hepaticojejunostomy was performed using a 5-0 PDS single-layer running suture. Thereafter, jejunojejunostomy was performed. A closed drainage catheter was placed around the resection plane of the liver and the hepaticojejunostomy. Abdominal closure was performed after hemostasis was achieved.

Despite abdominal drainage, the patient experienced a biloma that was treated by non-surgical approach with a percutaneous catheter insertion for drainage and spontaneous closure. Patient was discharged on postoperative day 12.

At the 2-months follow-up, the patient is asymptomatic with normal liver function tests and no recurrent lesions.

**Fig. 5:**
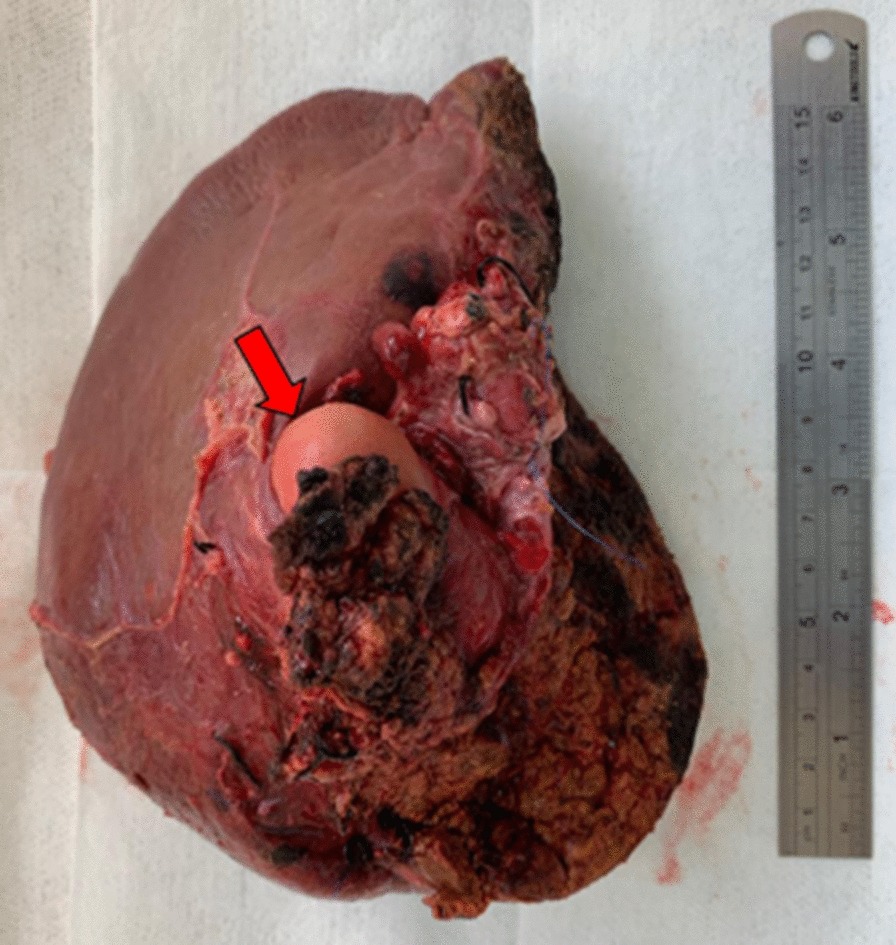
Surgical specimen. Cyst demonstrated by the red arrow

**Fig. 6:**
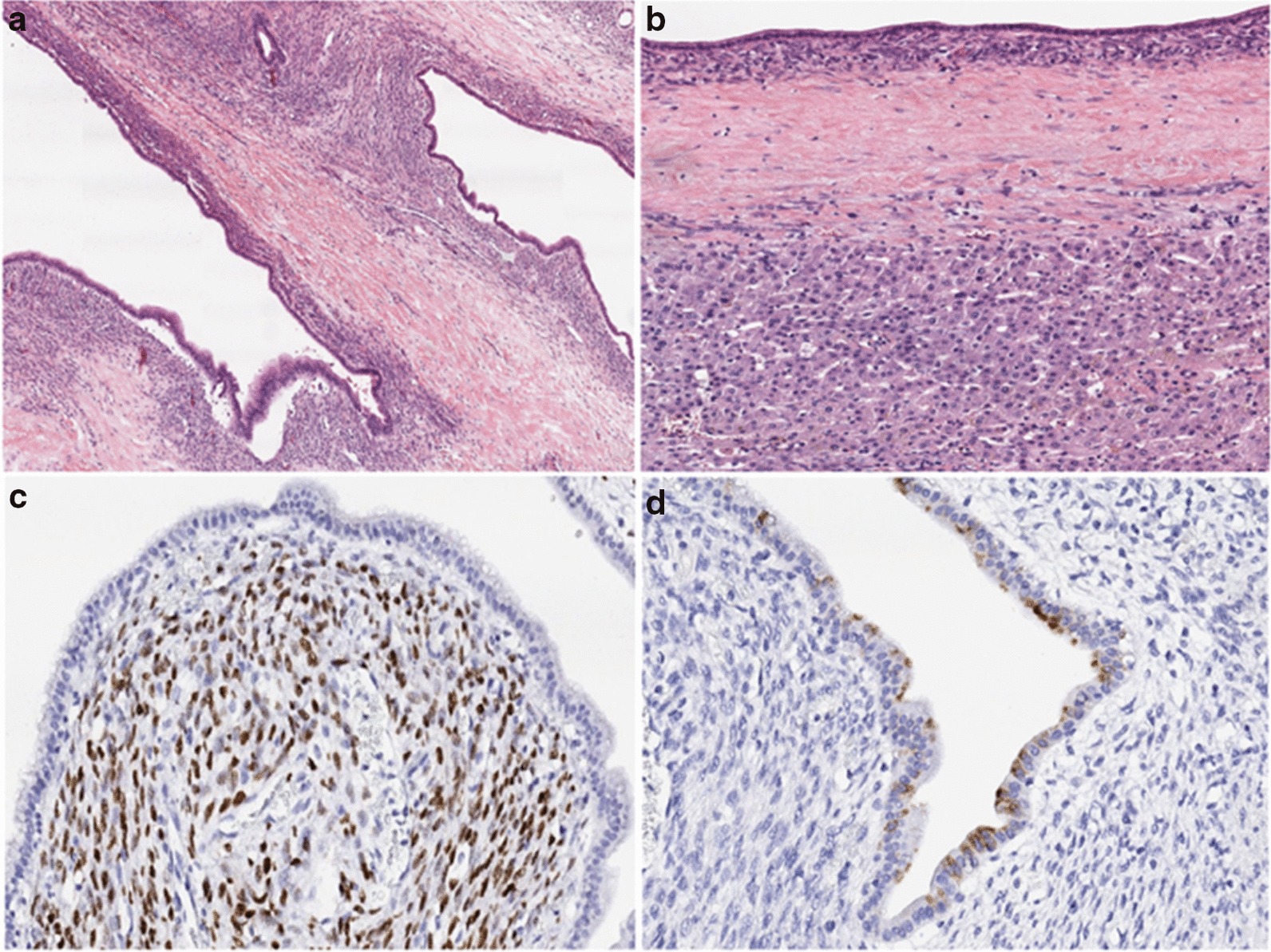
Mucinous cystic neoplasm of the liver. **a** The cysts are lined by flat, columnar, mucin-producing epithelium with low-grade dysplasia; **b** the cystic wall is lined with flat epithelium with a prominent underlying ovarian stroma. A thick fibrous wall separates the neoplasm from the adjacent liver parenchyma; **c** the stroma of the ovarian-type with positive immune-expression for the estrogen receptor; **d** some of the epithelial cells with positive immune-expression for gastric-type mucin (MUC5AC)

Surprisingly, histopathological examination of the resected specimen (Fig. 5) confirmed the diagnoses of low-grade MCN-L (Fig. 6). Histopathological findings are very characteristic of these neoplasms. The presence of ovarian stroma excludes other differential diagnoses of mucinous cystic lesions of the liver. The diagnosis was performed by a pathologist specialized in gastrointestinal and liver diseases. Double checking of diagnoses is routine in our pathology laboratory.

## Discussion and conclusion

The most recent WHO classification defined mucus-producing bile duct tumors of the liver into MCN-L and IPN-B [1]. MCN-L are rare cyst-forming epithelial neoplasm, usually showing no communication with the bile ducts, composed of cuboidal to columnar, variably mucin-producing epithelium, and associated with ovarian-type stroma. Mucinous cystic neoplasms need to be distinguished from other cystic liver lesions including simple cysts and cystic hamartomas. Recently, a unique biliary tumour named IPN-B has been included among biliary cystic tumours. IPN-B is considered as a biliary counterpart of intraductal papillary mucinous neoplasms of the pancreas and previous study revealed that cystic liver tumours with bile duct communication share characteristics with intraductal papillary neoplasms, but not MCN-L [6, 8]. Both types of mucinous cystic neoplasms may show malignant degeneration into cystadenocarcinomas [2].

Features of MCN-L on abdominal contrasted CT scan are hypodense lesions with internal septations which enhance with contrast [9]. Presence of irregular wall thickening, mural solid nodules, thick calcifications and papillary projections are suggestive of a cystadenocarcinoma. MCN-L tend to occur more often in middle-aged women, and are frequently located is central segments of the liver (typically S4) [6]. MCN-L is basically considered to be a non-invasive, that does not grow invasively but grows expansively [7]. Small lesions are usually asymptomatic while large cysts can present with abdominal pain or discomfort.

Peculiar features of the current case are the unusual location in right lobe, presentation with jaundice which is a rare clinical manifestation due to biliary obstruction [6], and communication with biliary duct. The preoperative diagnostic doubt unable to rule out a malignant intra-hepatic cholangiocarcinoma led to an individualized approach that required anatomic right hepatectomy with caudate lobe associated with complete bile duct resection and hilar lymph node dissection.

Unlike the case presented, this unique neoplasm is usually characterized by having no communication with the bile duct [10]. A previous study by Zen et al., revealed that cystic liver tumours with bile duct communication share characteristics with intraductal papillary neoplasms, but not mucinous cystic neoplasms [7]. Anand et al., described two rare cases of MCN-Ls with biliary communication and suggested that MCN-L should be subclassified into MCN-L with or without biliary communication [9].

Although in the past cystadenomas of the liver have been treated by marsupialisation, internal Roux-en-Y drainage, aspiration, sclerosis or partial resection, currently the best procedure is the complete surgical excision because of the potential of malignant degeneration [6]. The most commonly procedures performed are left or central hepatectomies due to major prevalence on central liver segments.

MCN-L is a rare pre-malignant tumor with some typical clinical and radiological features. Despite these characteristics, unusual presentations of MCN-L should be considered in the differential diagnosis of patients with cystic neoplasm and jaundice. Although biliary communication typically speaks against the diagnosis of MCN-L, it can occur in rare cases.

## Data Availability

Data sharing is not applicable to this article as no datasets were generated or analyzed during the current study.

## Abbreviations

BMI: Body mass index
BW: Body weight
CT: Computed tomography
FLR: Future liver remnant
IPN-B: Intraductal papillary neoplasm of the bile duct
MCN-L: Mucinous cyst neoplasm of the liver
MRI: Magnetic resonance imaging
TLV: Total liver volume
WHO: World Health Organization
